# New Inventions

**Published:** 1895-06

**Authors:** 


### NEW INVENTIONS.

A soft-tread horseshoe, consisting of a top-plate with two walls from the plate, the space between inner and outer wall hollow.

An aluminum horseshoe having finely-divided particles of hard metal distributed over and permanently imbedded in the wearing surface, curved and spiral strips of hardened metal imbedded in the shoe, with the wearing-edges on a level with the face of the shoe.

A horseshoe pad, composed of a plate and rubber heel, the pad being notched to receive the heels of the shoe.

A looped metal currycomb with serrated edges.

A rubber and canvas saddle-pad so arranged as to be capable of inflation.

A flexible collar-pad, with a central perforated ventilating-chamber on its under side.

A back-pad comprising a pad proper and a pad-back, with concave top, and transverse opening carried to the edges.

A new caponizing-instrument.

Automatic watering-trough for animals.

A circular spring currycomb consisting of a serrated endless band of metal.

A combination hopple with a breast-plate, a depending bracket thereon for leg-straps, to be secured to the limbs above the knees.

A horseshoe with removable heel and toe calkings.

A cushioned horseshoe, the cushion being elastic, of rubber, and so arranged as to fit in recesses of the shoe.

An improvement in milk-preparation, consisting of dividing whole milk into cream, milk without fat, and sugar of milk and water, each of which fluids is made to contain a definite and known percentage of milk-constituents, then recombining these constituents according to special formulas.

An animal-poke, with yoke for neck and nose-band, with a series of projecting arms.

A safety-rein bridle, with disks to close the nostrils when required to control or stop an animal.

A shouldered-calk for horseshoe, removable at will, made secure to the shoe by a double-ended screw passing through the calk.

A leg-spreader, made to bear against the inner faces of the legs, suspended over the shoulders from the withers.

Castrating-forceps, comprising a pair of oppositely arranged movable jaws, the lower jaw having a projection on the top roughened on its upper surface, with a knife cutting-blade set transversely to the jaws, the upper jaw to hold a medicated sponge in front of the knife.

A horseshoe-pad, for the posterior half of the foot, recessed into the wings of the shoes.

